# Restoration of Enzymatic Activity of Energy-Related Proteins in Rats with Traumatic Brain Injury Following Administration of Gamma-Glutamylcysteine Ethyl Ester

**DOI:** 10.3390/brainsci15101067

**Published:** 2025-09-30

**Authors:** Brittany Rice, Jonathan Overbay, Andrea Sebastian, Patrick G. Sullivan, Tanea T. Reed

**Affiliations:** 1 Department of Chemistry, Eastern Kentucky University, Richmond, KY 40475, USA; 2 Spinal Cord and Brain Injury Research Center, University of Kentucky, Lexington, KY 40506, USA

**Keywords:** traumatic brain injury, oxidative stress, glutathione, bioenergetics

## Abstract

**Background/Objectives:** Biochemical processes such as the glycolytic pathway and Kreb’s cycle are important in producing ATP for the brain. Without a sufficient supply of glucose for energy metabolism, the brain cannot efficiently regulate or coordinate the actions and reactions of the body. It is well documented that traumatic brain injury (TBI) is associated with reduced energy metabolism through the production of reactive oxygen/nitrogen species. Antioxidants, such as glutathione (GSH), have been shown to combat the deleterious effects of oxidation by scavenging ROS/RNS, inhibiting propagation, and removing neurotoxic byproducts. Gamma-glutamylcysteine ethyl ester (GCEE), an ethyl ester moiety of gamma-glutamylcysteine, exhibits antioxidant activity by increasing GSH production. This therapeutic has protective effects against oxidative stress through the elevation of glutathione. **Methods:** This study investigates the enzymatic activities of several key energy-related proteins that have been identified as nitrated in treated Wistar rats with moderate TBI. To test the hypothesis that the elevation of GSH production upon administration of GCEE will normalize enzymatic activity post-TBI, adult male Wistar rats were equally divided into three groups: sham, saline, and GCEE. Rats were treated with 150 mg/kg saline or GCEE at 30 and 60 min post-TBI. Upon sacrifice, brains were harvested and enzymatic activity was measured spectrophotometrically. **Results:** An increase in enzymatic activity upon GSH elevation via GCEE administration in several key enzymes was observed. **Conclusions:** GCEE is a potential therapeutic strategy to restore energy-related proteins in the brain post-TBI via GSH elevation.

## 1. Introduction

Traumatic brain injury (TBI) is a serious medical disorder and public health concern of significant proportions, as it is a leading cause of death and long-term disability in disproportionately affected heterogeneous populations worldwide, with an increasing burden of indirect and direct expenses associated with a lack of successful therapeutic strategies [1,2,3]. Higher incidences of traumatic brain injury are reported in individuals of minority descent and low socioeconomic status [4]. Studies confirm that this incidence and prevalence is increased in early childhood, late adolescence/early adulthood, and elderly populations. The high incidence in these groups may be attributed to a culmination of factors, such as recreational sports, motor vehicle accidents, reduced motor skills, and physical abuse, according to the National Center for Injury Prevention and Control at the Centers for Disease Control. A collection of studies demonstrate the association of gender differences with TBI, and females experience higher mortality and fatality rates from traumatic brain injury [5,6]. Evidence suggests that TBI can affect virtually everyone, considering risk factors and increasing incidence and prevalence rates.

Traumatic brain injury is a sudden spontaneous event in which brain dysfunction occurs as a result of an external force. The dysfunction experienced is a result of the external physical impact occurring at the site of injury, which is termed primary damage. Primary damage subsequently initiates immediate or delayed physiological disturbances, known as secondary damage, which collectively result in mitochondrial dysfunction. Mechanistically, this neurological disorder increases inflammation, neuronal damage, excitotoxicity, and cognitive impairment. After TBI, mitochondria experience an affluence of toxic radical species that decrease ATP production. In the pathological circumstances of traumatic brain injury, ATP production is often limited due to nitration of enzymes responsible for facilitating the biochemical reactions of cellular respiration. The scavenging of reactive oxygen/nitrogen species (ROS/RNS) is protective against oxidative stress; however, the antioxidant glutathione exists in the brain at lower concentrations than in other organs and is easily depleted post-TBI [7,8]. Strategies that increase glutathione concentrations post-brain injury, such as gamma-glutamylcysteine ethyl ester (GCEE), show therapeutic promise in protecting proteins against nitration and neurons against oxidative stress.

**Figure 1:**
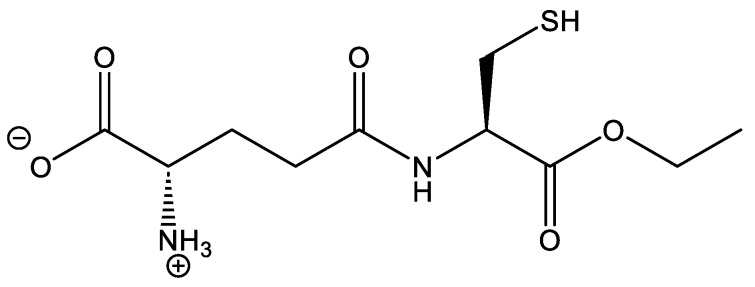
Structure of gamma glutamylcysteine ethyl ester (GCEE), a glutathione mimetic.

Proper brain function is dependent upon a sufficient supply of glucose for energy metabolism. Thus, biochemical processes involved in cellular respiration are important in producing ATP for the brain. Reduced energy metabolism and mitochondrial dysfunction are associated with traumatic brain injury, as studies have demonstrated that protein nitration is a consequence of TBI through the production of reactive oxygen and reactive nitrogen species. Antioxidants, such as glutathione (GSH), have been shown to combat the deleterious effects of oxidation by scavenging ROS/RNS, inhibiting propagation of lipid peroxyl radicals, and removing neurotoxic byproducts such as 4-hydroxynonenal, acrolein, and malondialdehyde [9,10]. Several experimental TBI treatments, including L-theanine [11], nicorandil [12], catalpol [13], and dexpaththenol [14], may upregulate glutathione levels and promote glutathione peroxidase activity, thereby reducing oxidative stress. Gamma glutamylcysteine ethyl ester (GCEE), an ethyl ester moiety of gamma-glutamylcysteine, exhibits antioxidant activity by increasing glutathione production. The structure is shown in Figure 1. The introduction of the ethyl ester moiety allows for easier transport across the blood–brain barrier, making it a more effective treatment than the tripeptide glutathione [15]. GCEE has been shown to cross the plasma membrane in neurons and as well as endothelial cells. Previous studies have demonstrated that the administration of GCEE following TBI has protective effects against protein nitration through the elevation of glutathione [8,16,17].

Reduced metabolism is a common occurrence in multiple neurodegenerative disorders, as seen in Alzheimer’s [18], Parkinson’s [18], and Huntington’s disease [19]. Traumatic brain injury is a risk factor for Alzheimer’s disease, therefore a link to increased neurodegeneration and secondary sequalae is associated with it. Reductions in energy metabolism and protein catabolism have been reported post-TBI. To glean a deeper understanding of the bioenergetics associated with moderate traumatic brain injury and glutathione elevation, we assessed the efficacy of GCEE as a therapeutic strategy in the overall restoration of brain energy metabolism via glutathione elevation following the onset of injury in this study. From brain homogenate, the enzymatic activity of energy-related proteins that had been identified as nitrated in moderate TBI were studied. Several energy-related enzymes were identified as nitrated in rats that had undergone a moderate traumatic brain injury and had been treated with GCEE or saline [20]. The key enzymes evaluated in this study were aspartate aminotransferase (AST), ATP synthase, cytochrome C oxidase (CCO), creatine kinase (CK), enolase (ENO), glyceraldehyde-3-phosphate dehydrogenase (GAPDH), lactate dehydrogenase (LDH), malate dehydrogenase (MDH), and pyruvate kinase (PK). Although enzymatic activity has been investigated in traumatic brain injury, this has not been investigated using a glutathione mimetic post-therapeutic strategy. These energy-related enzymes were assayed at various time points to determine outcomes at different time points. This work bolsters previous work in our lab which demonstrated reduced oxidative stress at longer timepoints [20]. The outcomes provide new insight into metabolic activity in traumatic brain injury at these time points and its correlation, if any, to other neurological disorders such as AD, HD, and PD. The results demonstrate an increase in enzymatic activity upon GSH elevation via GCEE administration in several key enzymes, including glyceraldehyde-3-phosphate dehydrogenase, pyruvate kinase, lactate dehydrogenase, aspartate aminotransferase, malate dehydrogenase, cytochrome C oxidase, and ATP synthase, thereby indicating GCEE is a potential therapeutic strategy to restore energy-related proteins in the brain post-TBI via GSH elevation.

### 1.1. Aspartate Aminotransferase

**Figure 2:**
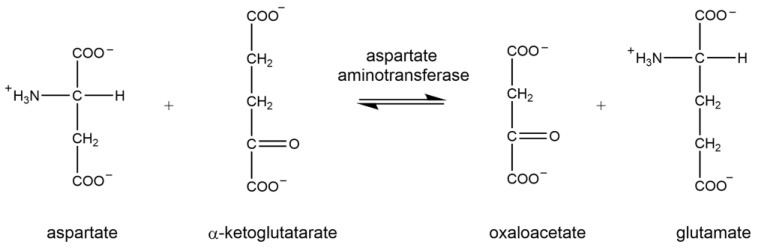
Enzymatic reaction of aspartate aminotransferase.

Aspartate aminotransferase (AST) facilitates and accelerates the reversible conversion of aspartate and α-ketoglutarate to oxaloacetate and glutamate, respectively (Figure 2). Brain injury results in the elevation of AST levels in serum. Measurement of AST activity following brain injury via coupled kinetic photometric analysis offers insights regarding the role of amino acid metabolism in the TCA cycle.

### 1.2. ATP Synthase Assay

**Figure 3:**
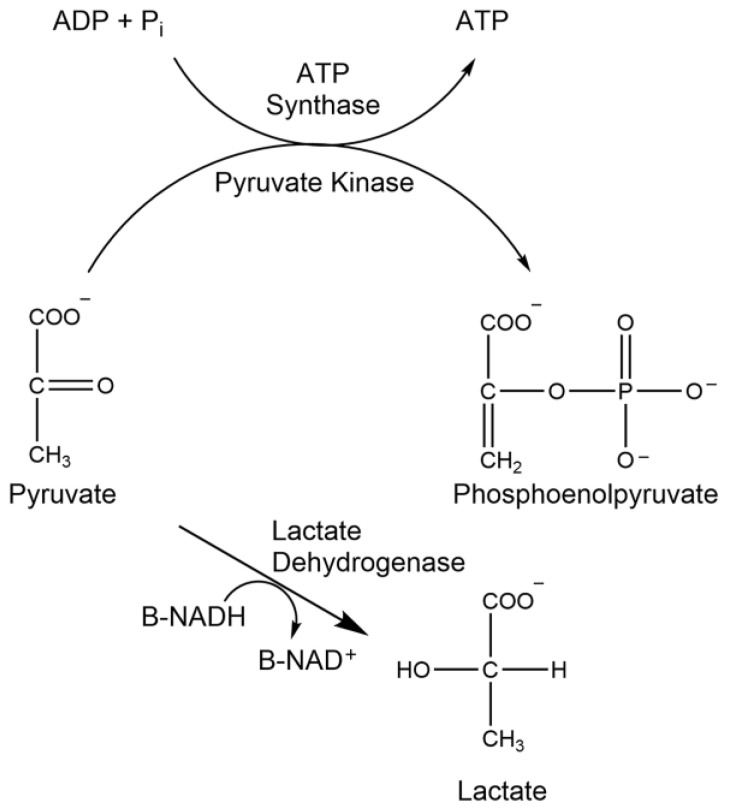
Enzymatic reaction of ATP synthase.

ATP synthase is a membrane protein that plays a pivotal role in energy metabolism, as it is responsible for catalyzing the phosphorylation of ADP to ATP (Figure 3). A deficiency or decrease in ATP synthase activity results in impaired ATP production, which has been observed in multiple neurodegenerative disorders [21,22,23].

### 1.3. Cytochrome C Oxidase

**Figure 4:**
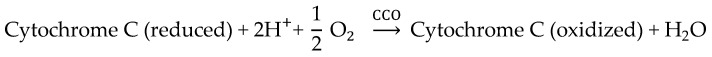
Enzymatic reaction of cytochrome C oxidase.

Cytochrome C oxidase (CCO) functions to provide energy to the cell via coupling electron transport and oxidative phosphorylation (Figure 4) in Complex IV of the respiratory chain. Dysfunction in this mitochondrial enzyme may result in an increase in oxidative stress [24] and lead to Leigh’s syndrome, which can be associated with overall mitochondrial dysfunction [25].

### 1.4. Creatine Kinase

**Figure 5:**
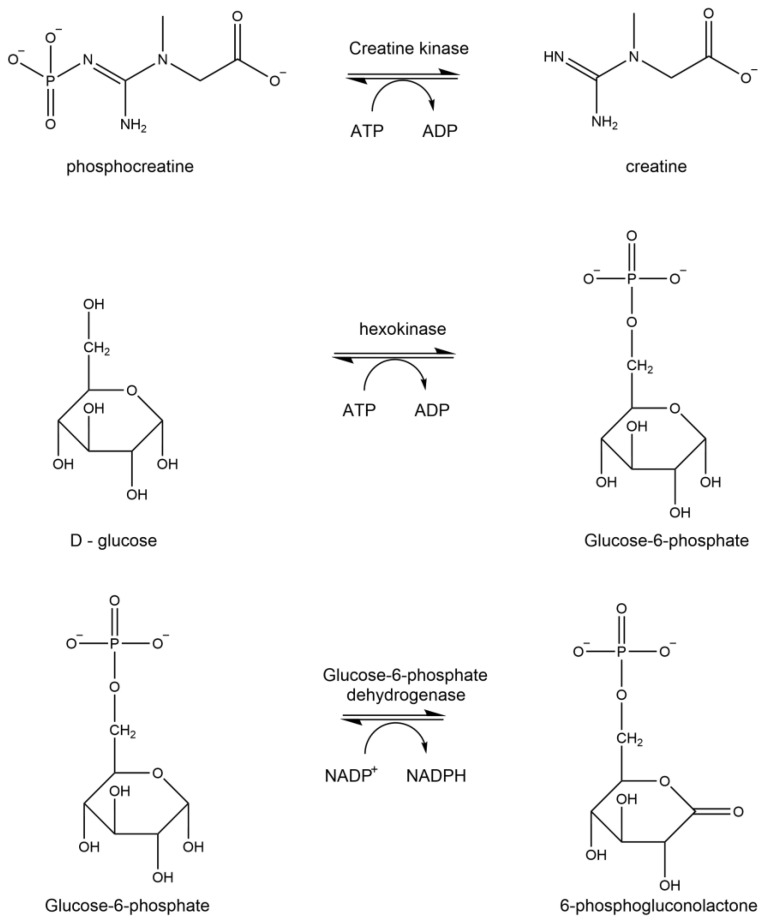
Enzymatic reaction of creatine kinase.

Creatine kinase (CK) is responsible for the production of energy and formation of the high energy biomolecule phosphocreatine (Figure 5). There is a strong correlation between TBI severity and creatine kinase levels, making it a plausible biomarker for traumatic brain injury [26,27,28]. Injury causes an elevation in this enzyme that can lead to dysfunction and reduced glucose metabolism, as evidenced in exercise, as it is a measure of muscle damage [29]. Due to the reduced bioenergetics associated with modified creatine kinase, this enzyme has been found to be altered in early-stage Alzheimer’s disease [30,31] and traumatic brain injury [26,32].

### 1.5. Enolase

**Figure 6:**
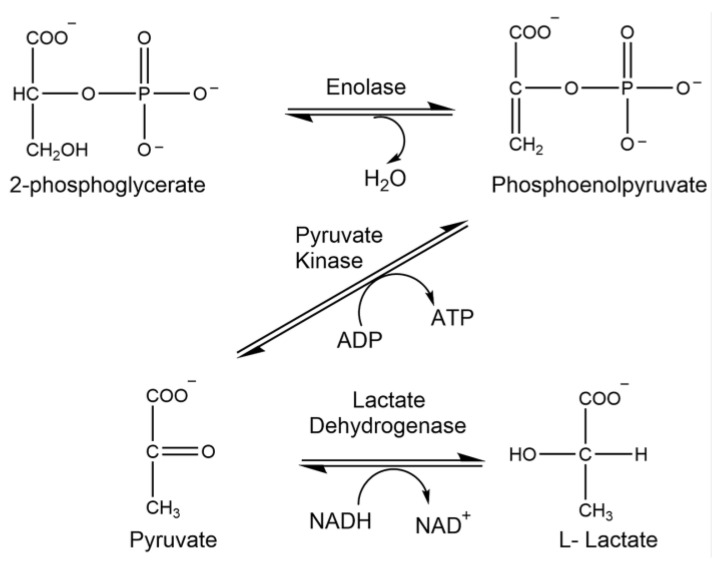
Enzymatic reaction of enolase.

Enolase (ENO) is the penultimate enzyme in glycolysis (Figure 6). Reduced glucose metabolism and enzymatic dysfunction via protein modification has been observed in neurodegenerative disorders and severe traumatic brain injury [24,33]. The consequences yield a reduction in ATP levels, which can have a global impact on overall memory and health.

### 1.6. Glyceraldehyde-3-Phosphate Dehydrogenase

**Figure 7:**
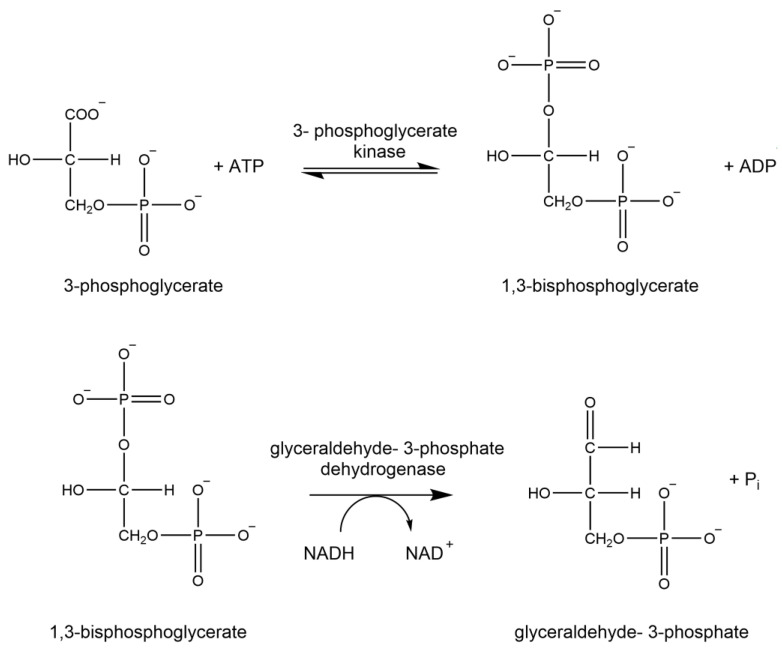
Enzymatic reaction of glyceraldehyde-3-phosphate dehydrogenase.

Glyceraldehyde-3-phosphate dehydrogenase, as observed in Figure 7, is the oxidoreductase responsible for ATP production in glycolysis. Apart from its role in glucose metabolism, GAPDH acts as an intracellular sensor of oxidative stress in the initiation of apoptosis and is involved in membrane trafficking, nuclear translocation, and immunomodulation [34,35,36]. Thus the quantitation of glyceraldehyde-3-phosphate dehydrogenase provides insight into normal cellular physiology and the detection of disease.

### 1.7. Lactate Dehydrogenase

**Figure 8:**
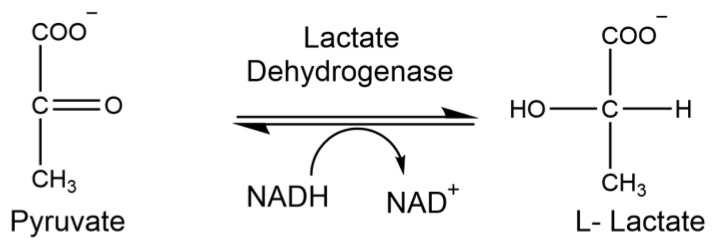
Enzymatic reaction of lactate dehydrogenase.

Lactate dehydrogenase (LDH) is an oxidoreductase responsible for the reduction of pyruvate to lactate (Figure 8). LDH levels are elevated and released into the bloodstream upon injury, disease, or exposure to toxic materials. Thus, clinical quantification of this enzyme is used to investigate pathological conditions and assess tissue damage and toxicity.

### 1.8. Malate Dehydrogenase

**Figure 9:**
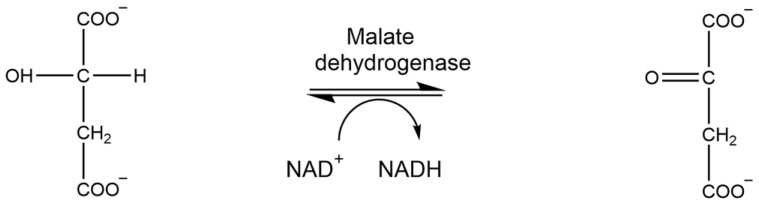
Enzymatic reaction of malate dehydrogenase.

Malate dehydrogenase (MDH) is an oxidoreductase that participates in the reversible catalysis of the conversion of L-malate to oxaloacetate using NAD+ (Figure 9). Research indicates increased MDH activity in Alzheimer’s disease may be the consequence of oxidative stress [37]. Thus, quantifying the activity of MDH post-TBI provides insight into the correlation between oxidative stress and neurodegenerative diseases.

### 1.9. Pyruvate Kinase

**Figure 10:**
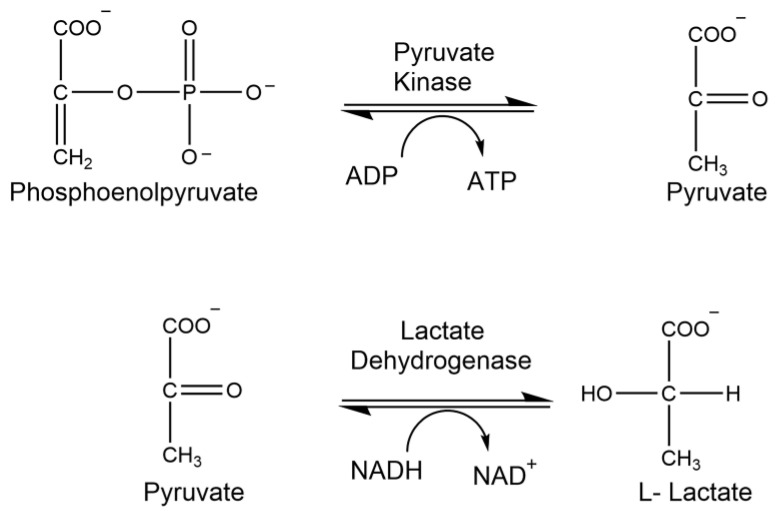
Enzymatic reaction of pyruvate kinase.

Pyruvate kinase (PK) is an essential enzyme in glucose metabolism as it catalyzes the conversion of phosphoenolpyruvate (PEP) into pyruvate and assists in the production of ATP in glycolysis (Figure 10). Pyruvate produced from this reaction serves as an intermediate, connecting glycolysis and the TCA cycle. Thus the strict regulation of PK activity is of immense importance in overall cellular metabolism. Defects in pyruvate kinase activity impede glycolysis, while the absence of PK causes the development of life-threatening hemolytic anemia [38]. This enzyme is energetically linked to the antioxidant glutathione, as glutathione reductase deficiency increases levels of oxidized glutathione, thereby reducing antioxidant defense [39].

Evidence demonstrates that GCEE reduces oxidative stress via the biomarkers of protein carbonyls and 3-nitrotyrosine. This work is a deeper analysis of the specific enzymatic activity of the aforementioned proteins, which was found to be oxidatively modified in moderate traumatic brain injury. Many of these enzymes are also found in other neurodegenerative disorders such as Alzheimer’s disease, Huntington’s disease, and Parkinson’s disease [40,41,42,43]. This is the first paper to compare enzymatic assays of these enzymes in TBI, AD, HD, and PD, therefore making it novel.

## 2. Materials and Methods

### 2.1. Chemicals

All chemicals, unless otherwise indicated, were obtained from Sigma Aldrich (St. Louis, MO, USA) and were of the highest purity. GCEE was purchased from Bachem (Torrance, CA, USA).

### 2.2. Surgical Methods

The surgical procedures and drug administration protocols described below are in compliance with and have been approved by the University of Kentucky Institutional Animal Care and Use Committee and are consistent with all animal care procedures established by the U.S. Public Health and Service Policy on Humane Care and Use of Laboratory Animals (protocol number D16-00217).

Thirty adult male 5-month-old Wistar rats (Harlan Laboratories, Indianapolis, IN, USA) were used, weighing 300–350 g. They were equally divided into three experimental groups: sham, saline, and GCEE. Six animals were chosen for each treatment group based on previous studies showing that this number demonstrated statistical significance. Animals were shaved and anesthetized using isoflurane (3.0%) prior to being placed in a stereotaxic frame (David Kopf Instruments, Tujunga, CA, USA) for surgery. Surgery was completed and conducted in the same fashion as previously described by Sullivan [44]. Sham animals were excluded from the injury but received a craniotomy (*n* = 6). Post craniotomy, each Wistar rat had a 4 mm dental cement seal placed on the craniotomy site. The rats were then placed on warming mats to prevent hypothermia until they regained consciousness. The rats were given 150 mg/kg body weight of GCEE 30 or 60 min post-injury (*n* = 6). Injections were given intraperitoneally based on prior research in which improvement with post-neurosteroidal treatment was observed. Another group was given saline at 30 or 60 min post-injury, with a concentration of 150 mg/kg body weight (*n* = 6). A summary of the experimental design can be seen in Table 1.

Concentrations of GCEE and saline were determined from previous studies [17]. No adverse effects were observed after GCEE administration in this study. All rats were then sacrificed 24 h post-injury with immediate removal and freezing of the whole brain at −80 °C. The time period of 24 h was chosen due to the maximum decrease in glutathione levels as well as enzymatic activity occurring during it [16]. All water used in this procedure was distilled and deionized.

Upon sacrifice, the whole brain was harvested, suspended, sonicated, and homogenized in 10 mM HEPES buffer (pH 7.4) containing 137 mM NaCl, 4.6 mM KCl, 1.1 mM KH_2_PO_4_, 0.1 mM EDTA, and 0.6 mM MgSO_4_ along with protease inhibitors: leupeptin (0.5 mg/mL), pepstatin (0.7 µg/mL), type II S soybean trypsin inhibitor (0.5 µg/mL), and phenylmethylsulfonyl fluoride (PMSF; 40 µg/mL). Brain homogenates were centrifuged to remove debris at 14,000× *g* for 10 min, and supernatant was collected.

All enzymatic assays were completed using standard protocols from Sigma Aldrich [45].

### 2.3. Analysis of Aspartate Aminotransferase Activity

The activity of aspartate aminotransferase (AST) was measured spectrophotometrically by malate dehydrogenase coupled spectrophotometric assay. The standard reaction mixture contained 134 mM L-aspartic acid (pH 7.4), 6.64 mM α-ketoglutaric acid, 240 mM β-NADH, 5 units LDH, 1.25 units MDH, and 50 mM Na_3_PO_4_. Five microliters of AST enzyme or brain sample at room temperature initiated the reaction. One unit of enzyme oxidizes one micromole of β-NADH per minute. The assay was carried out in a microtiter plate reader at 340 nm (Bio-Tek Instrument Inc., Winooski, VT, USA).

### 2.4. Analysis of ATP Synthase Activity

Mitochondrial ATP synthase activity was measured spectrophotometrically at 340 nm by coupling the production of ADP to the oxidation of NADPH via the pyruvate kinase (PK) and lactate dehydrogenase reaction (coupled assay) as described [46]. The reaction mixture (0.2 mL final volume) contained 100 mM Tris (pH 8.0), 4 mM Mg-ATP, 2 mM MgCl_2_, 50 mM KCl, 0.2 mM EDTA, 0.23 mM NADH, 1 mM phosphoenolpyruvate, 1.4 unit PK, 1.4 unit lactate dehydrogenase, and about 25–50 μg protein (brain homogenate), and was assayed at 30 °C. The assay was carried out in a microtiter plate reader (Bio-Tek Instrument Inc., Winooski, VT, USA).

### 2.5. Analysis of Cytochrome C Oxidase Activity

Prior to performing this assay, a stock solution containing 10 milligrams per milliliter of cytochrome C was reduced with excess sodium L-ascorbate and then dialyzed before use. The reaction mixture contained 2 µg of reduced cytochrome C and 10 mM KH_2_PO_4_ (pH 7). The cytochrome C and ascorbic acid solution was then placed in a cellophane membrane and dialyzed in 10 mM KH_2_PO_4_ buffer for 24 h in an effort to remove excess ascorbic acid from the dialysate (cytochrome C and ascorbic acid solution). The cytochrome C oxidase enzyme was prepared in 250 mM sucrose solution with 1% Tween 80 (*v*/*v*). A complete reaction mixture along with the addition of 100 mM K_3_Fe(CN)_6_ served as the blank. All reagents utilized in this assay were prepared in 100 mM KH_2_PO_4_ buffer (pH 7.4 at 37 °C). The reaction was started by the addition of 6.7 µL of cytochrome C or sample and equilibrated at 37 °C. One unit of cytochrome c oxidase oxidizes one micromole of reduced cytochrome C to oxidized cytochrome C per minute. The assay was carried out in a microtiter plate reader at 550 nm (Bio-Tek Instrument Inc., Winooski, VT, USA).

### 2.6. Analysis of Creatine Kinase Activity

The enzyme activity of creating kinase was determined at 30 °C using a series of three reactions (Figure 5). The standard reaction mixture contained 250 mM glycine–glycine buffer with 0.10% BSA (pH 7.4), 400 mM phosphocreatine solution, 40 mM ADP solution, 1 M D-glucose solution, 20 mM β-NADP, and 300 mM Mg(C_2_H_3_O_2_)_2_. The solution was adjusted to pH 7.4. Ten microliters of both hexokinase (300 units/mL) and glucose 6-phosphate dehydrogenase (10 units/mL) prepared in water were used to facilitate the enzyme assay. One unit of activity is the amount of enzyme catalyzing the oxidation of 1 μmol NADH/min under the above conditions The assay was carried out in a microtiter plate reader at 340 nm (Bio-Tek Instrument Inc., Winooski, VT, USA).

### 2.7. Analysis of Enolase Activity

The enzyme activity of enolase was determined using a series of three reactions (Figure 6). The standard reaction mixture contained 100 mM triethanolamine buffer (pH 7.4), 56 mM 2-phosphoglycerate, 7 mM β-NADH, 500 mM MgSO_4_, 2M KCl, and 20 mM ADP (pH 7.4). Following this, 3.70% (*v*/*v*) pyruvate kinase/lactic dehydrogenase enzyme mix (Sigma Aldrich #P-0294) was added. Once incubated at 25 °C for five minutes, five microliters of sample or buffer was used to initiate the reaction. One unit of activity is the amount of enzyme catalyzing the oxidation of 1 μmol NADH/min under the above conditions. The assay was carried out in a microtiter plate reader at 340 nm (Bio-Tek Instrument Inc., Winooski, VT, USA).

### 2.8. Analysis of Glyceraldehyde-3-Phosphate Dehydrogenase Activity

Glyceraldehyde-3-phosphate dehydrogenase enzyme activity was measured through coupled spectrophotometric assay at 340 nm by the production of NAD+ from NADH through the conversion of glycerate to 1,3 biphosphate by glyceraldehyde-3-phosphate dehydrogenase. The reaction mixture (0.2 ml final volume) contained 100 mM triethanolamine hydrochloride (pH 7.6), 100 mM D(−)3-phosphoglyceric acid (cyclohexylammonium) salt, 200 mM L-cysteine hydrochloride monohydrate, 100 mM MgSO_4_, 7 mM β-NADH, 34 mM ATP, and 200 units 3-phosphoglyceric phosphokinase. The addition of 6.8 µL of GAPDH or brain sample initiated the reaction. The assay was carried out at room temperature in a microplate reader (Bio-Tek Instrument Inc., Winooski, VT, USA).

### 2.9. Analysis of Lactate Dehydrogenase Activity

Lactate dehydrogenase (LDH) enzyme activity was measured spectrophotometrically at 340 nm by the production of NAD+ from NADH through the conversion of pyruvate to lactate by lactate dehydrogenase. The reaction mixture (0.2 mL final volume) contained 0.13 mM β-NADH (pH 7.3) in 100 mM sodium phosphate buffer (pH 7.5), and 69 mM pyruvate. Five microliters of sample were used for this reaction. The assay was carried out at 37 °C in a microtiter plate reader (Bio-Tek Instrument Inc., Winooski, VT, USA).

### 2.10. Analysis of Malate Dehydrogenase Activity

Malate dehydrogenase activity was determined spectrophotometrically by measuring the decrease in NADH absorbance at 340 nm for 15 min. The reaction mixture contained 100 mM of KH_2_PO_4_ (pH 7.4), 6 mM oxaloacetic acid, and 3.75 Mm β-NADH. Five microliters of MDH or sample to reaction mixture initiated the assay. One unit of malate dehydrogenase catalyzes the conversion of one micromole of oxaloacetic acid and β-NADH to L-malate and β-NAD+ per minute. The assay was carried out in a microtiter plate reader at 340 nm (Bio-Tek Instrument Inc., Winooski, VT, USA).

### 2.11. Analysis of Pyruvate Kinase Activity

The enzyme activity of pyruvate kinase was determined at 37 °C by lactate dehydrogenase-coupled spectrophotometric assay [47]. The standard reaction mixture contained 100 mM Tris pH 8.0, 100 mM KCl, 10 mM MgCl_2_, 0.5 mM EDTA, 0.2 mM NADH, 10 μg LDH, 10 mM phosphoenolpyruvate, and 1.5 mM ADP in a final volume of 1 mL. The reaction was started by adding enzyme solution (0.5–1 μg). One unit of activity is the amount of enzyme catalyzing the oxidation of 1 μmol NADH/min under the above conditions. The assay was carried out in a microtiter plate reader at 340 nm (Bio-Tek Instrument Inc., Winooski, VT, USA).

### 2.12. Specific Activity

The calculation for specific activity was determined from protocols given by Sigma-Aldrich (St. Louis, MO, USA). By using the Beer–Lambert law and the fact that NADH_340_ has an extinction coefficient of 6.220 L mmol^−1^ cm^−1^, we can determine the concentration of NADH. Enzyme activity is defined as the rate of reaction multiplied by the sample volume. Specific enzyme activity is a measure of enzyme efficiency and is the enzyme activity divided by the mass.

### 2.13. Statistical Analysis

All statistical analyses were performed using GraphPad PRISM software (version 10.4.1) and Student’s *t*-test. The significance of each result was confirmed by calculation of the *p*-value by two-tailed Student’s *t*-tests (*p*-values <0.05). Error bars were calculated as SEM.

## 3. Results

### 3.1. Enzymatic Analysis

#### 3.1.1. Transferase Enzymes (AST and CK Activities)

**Figure 11:**
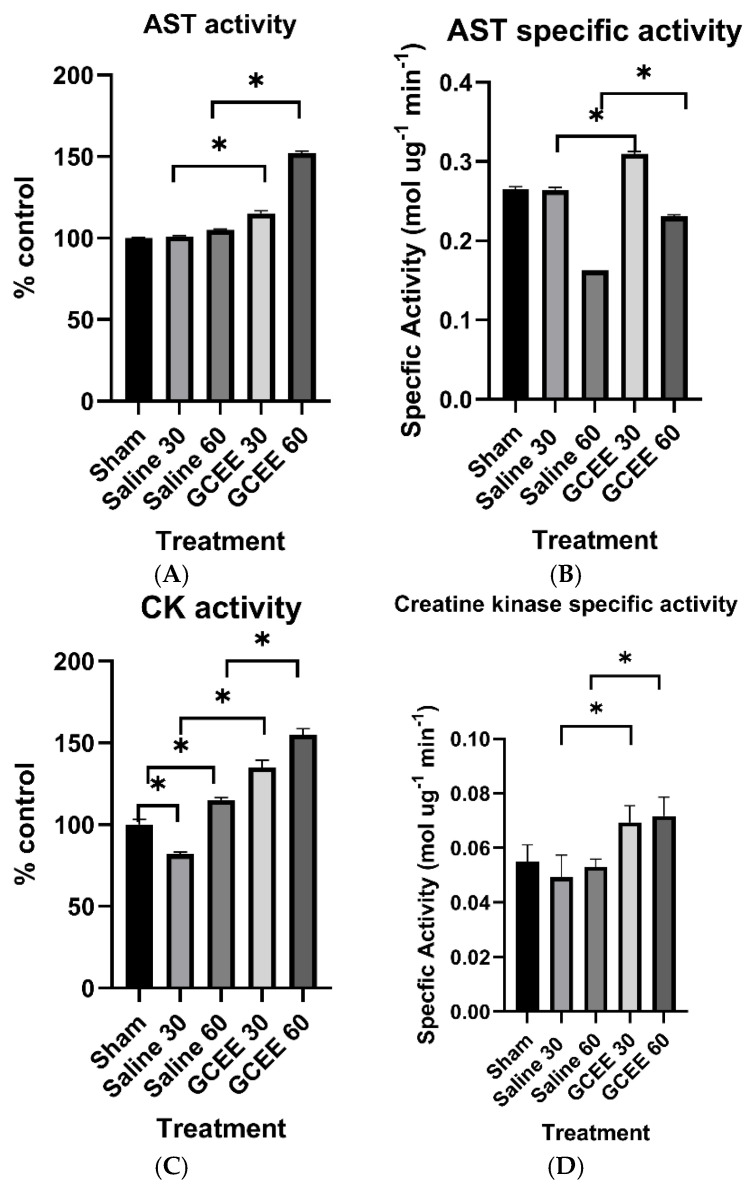
(**A**) Activity of aspartate aminotransferase at 30 and 60 min post traumatic brain injury. *n* = 6. Error is reported in SEM (* *p* < 0.05). (**B**) Specific activity of AST at 30 and 60 min in brain samples treated with saline and GCEE, respectively, *n* = 6. Error is reported in SEM (* *p*-value < 0.05). (**C**) Activity of creatine kinase at 30 and 60 min post traumatic brain injury. *n* = 6. Error is reported in SEM (* *p*-value < 0.05). (**D**) Specific activity of creatine kinase at 30 and 60 min in brain samples treated with saline and GCEE, respectively, *n* = 6. Error is reported in SEM (* *p*-value < 0.05).

Measurements of aspartate aminotransferase activity in rats with traumatic brain injury demonstrated a slight increase following saline administration 30 min after injury compared to sham rats. The administration of GCEE post-TBI significantly increased activity compared to sham and saline-treated animals at 30 min post-injury. Observations of the effect of time of treatment demonstrated significance, where administration of saline and GCEE 60 min after injury increased enzyme activity compared to administration at 30 min post-injury, and the increases caused by saline and GCEE were similar but statistically significant (Figure 11A). GCEE administration following TBI led to an increase in the enzyme activity of creatine kinase compared to in the sham and saline groups (Figure 11C), which was statistically significant (*p* < 0.05). The specific activities of these enzymes have been calculated and displayed in Figure 11B,D.

#### 3.1.2. Oxidative Phosphorylation Proteins (ATP Synthase and CCO Activities)

**Figure 12:**
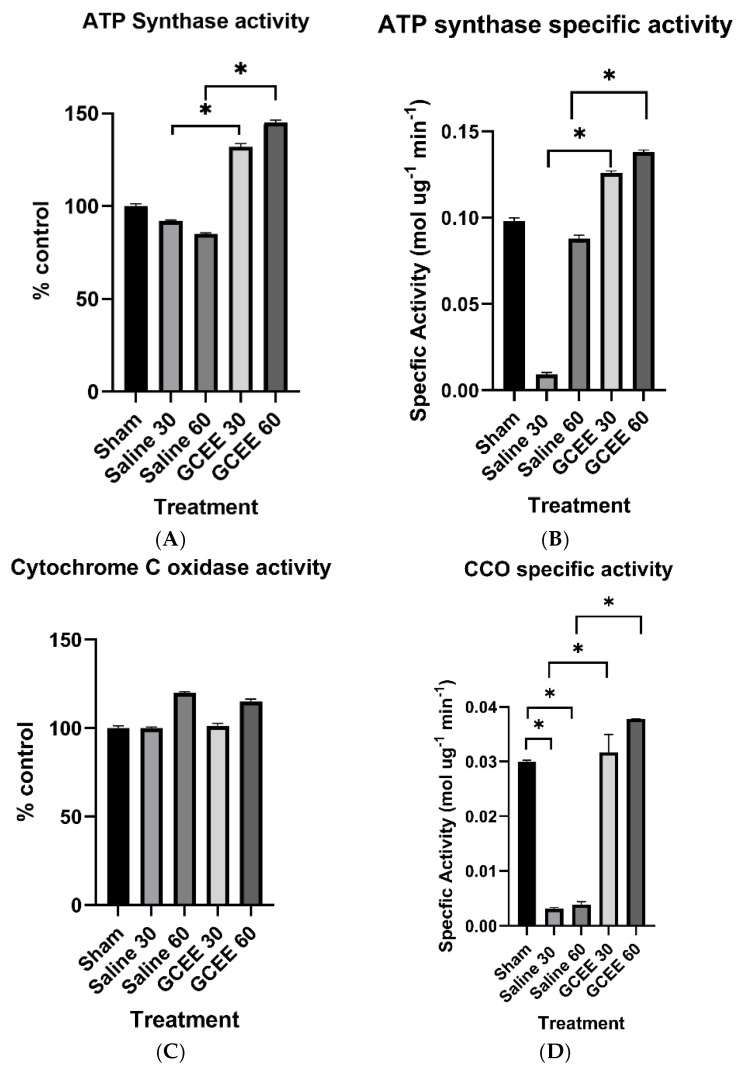
(**A**) Activity of ATP synthase at 30 and 60 min post traumatic brain injury. *n* = 6. Error is reported in SEM (* *p*-value < 0.05). (**B**) Specific activity of ATP synthase at 30 and 60 min in brain samples treated with saline and GCEE, respectively. *n* = 6. Error is reported in SEM (* *p*-value < 0.05). (**C**) Activity of cytochrome C oxidase at 30 and 60 min post traumatic brain injury. *n* = 6. Error is reported in SEM. (**D**) Specific activity of CCO at 30 and 60 min in brain samples treated with saline and GCEE, respectively. *n* = 6. Error is reported in SEM (* *p*-value < 0.05).

Reductions in the activity of ATP synthase were observed upon the administration of saline compared to the control group (Figure 12A). An increase in enzymatic activity was observed after the administration of GCEE at both time points compared to saline-treated and sham animals. These differences did achieve statistical significance (*p* < 0.05).

For cytochrome C oxidase, activity upon administration of saline and GCEE in rats with traumatic brain injury was determined to be not statistically significant (*p* > 0.05), with the greatest increase in activity observed 60 min post-injury (Figure 12C). The specific activities of ATP synthase and cytochrome C oxidase are shown in Figure 12B and Figure 12D, respectively.

#### 3.1.3. Glycolytic Enzymes (ENO, GAPDH, and PK Activities)

**Figure 13:**
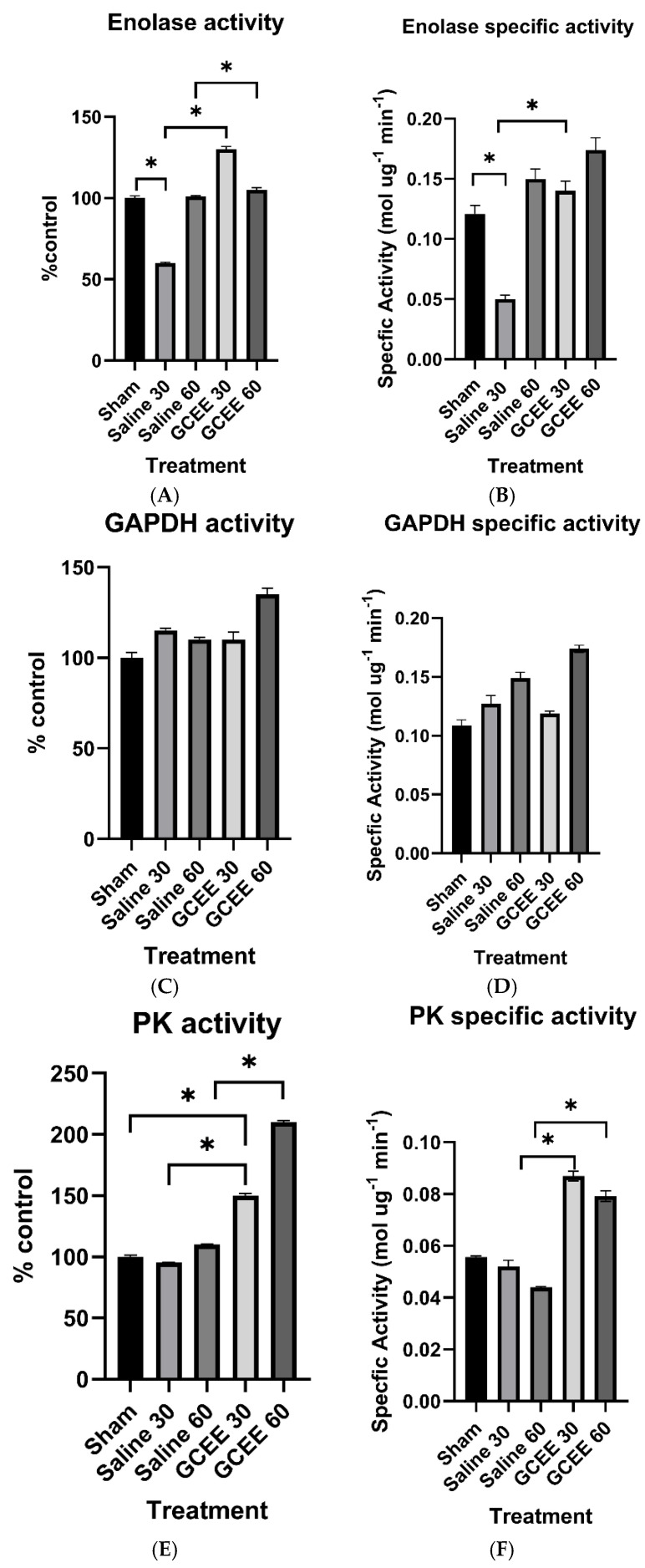
(**A**) Activity of enolase at 30 and 60 min post traumatic brain injury. *n* = 6. Error is reported in SEM (* *p*-value < 0.05). (**B**) Specific activity of enolase at 30 and 60 min in brain samples treated with saline and GCEE, respectively. Bars represent mean ± SEM (* *p* < 0.05). *n* = 6 for each group. (**C**) Activity of glyceraldehyde-3-phosphate dehydrogenase at 30 and 60 min post traumatic brain injury. *n* = 6. Error is reported in SEM. (**D**) Specific activity of GAPDH 30 and 60 min in brain samples treated with saline and GCEE, respectively. *n* = 6. Error is reported in SEM. (**E**) Activity of pyruvate kinase at 30 and 60 min post traumatic brain injury. *n* = 6. Error is reported in SEM (* *p*-value < 0.05). (**F**) Specific activity of pyruvate kinase at 30 and 60 min in brain samples treated with saline and GCEE, respectively. *n* = 6. Error is reported in SEM (* *p*-value < 0.05).

The administration of saline and GCEE following TBI caused an increase in the enzyme activity of enolase compared to the sham and saline groups, which was statistically significant at both time points (*p* < 0.05). The increase was more pronounced at the 30 min time point compared to the 60 min mark (Figure 13A).

The administration of saline and GCEE following TBI caused an increase in the enzyme activity of glyceraldehyde-3-phosphate dehydrogenase compared to the sham group, but was not statistically significant (*p* > 0.05). The administration of saline 30 min post-injury had a greater effect on the rats in comparison to those treated with saline 60 min post-TBI; whereas the administration of GCEE 60 min post-TBI had a greater effect on the rats in comparison to those treated with GCEE 30 min post-injury. However, the differences in treatment time were marginally statistically significant compared to controls (*p* = 0.0593). This is demonstrated in Figure 13C.

The catalytic activity of PK using the pyruvate kinase assay was determined. A non-significant reduction in activity was observed following the administration of saline 30 min after injury compared to sham, but increased within 60 min (Figure 13E). PK activity significantly increased post-injury following the administration of GCEE 30 and 60 min post-TBI compared to sham and saline treated animals (*p* < 0.05), with an elevation in activity at 60 min. Specific activities were calculated for ENO, GAPDH, and PK. These are shown in Figure 13B, Figure 13D, and Figure 13F, respectively.

#### 3.1.4. Dehydrogenases (LDH and MDH Activities)

**Figure 14:**
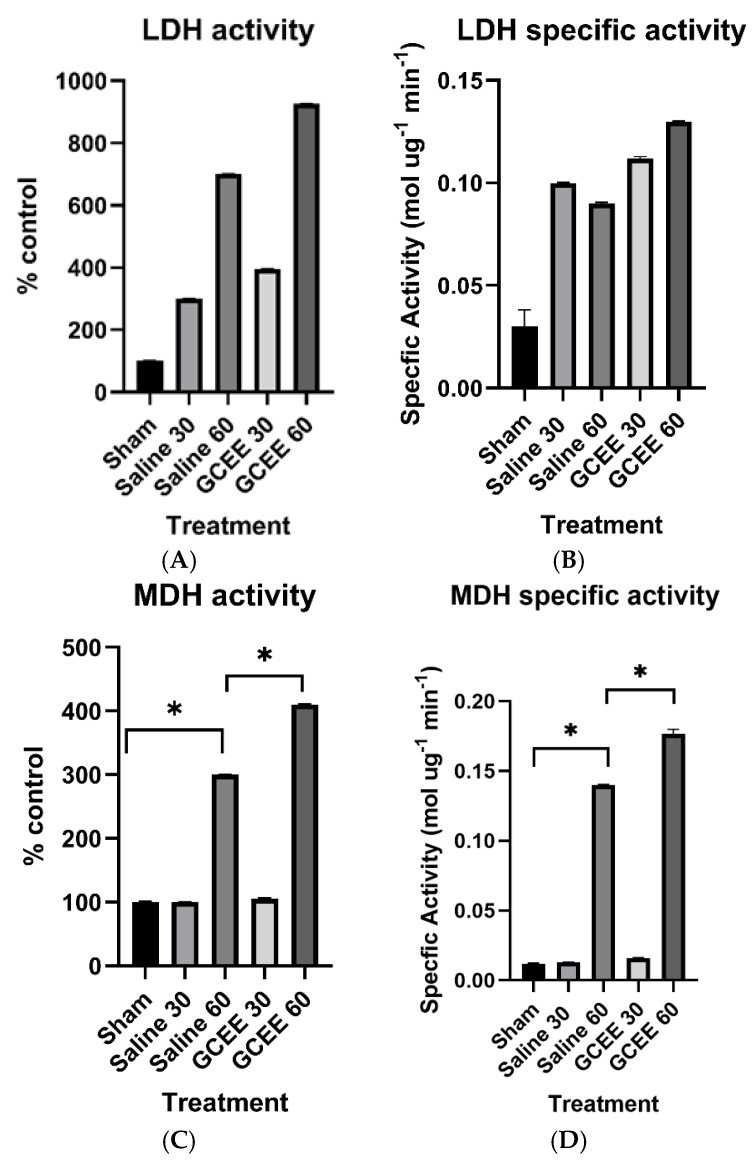
(**A**) Enzymatic activity of lactate dehydrogenase at 30 and 60 min post traumatic brain injury. *n* = 6. Error is reported in SEM. (**B**) Specific activity of LDH at 30 and 60 min in brain samples treated with saline and GCEE, respectively. *n* = 6. Error is reported in SEM. (**C**) Activity of malate dehydrogenase at 30 and 60 min post traumatic brain injury. *n* = 6. Error is reported in SEM (* *p* < 0.05). (**D**) Specific activity of MDH at 30 and 60 min in brain samples treated with saline and GCEE, respectively. *n* = 6. Error is reported in SEM (* *p*-value < 0.05).

The administration of saline and GCEE at 30 and 60 min post-injury increased LDH activity compared to the control. However, this was not a significant increase (*p* > 0.05). Although the administration of both saline and GCEE 60 min post-injury had a more pronounced effect on rats in comparison to those treated at 30 min post-injury, the elevation in activity was nonsignificant, as shown in Figure 14A (*p* > 0.05).

The results indicate the administration of the vehicle (saline) and drug significantly increased activity compared to the sham group (*p* < 0.05). A more robust significant increase in activity was observed following the administration of saline and GCEE 60 min post-TBI, in comparison to administration at 30 min (*p* < 0.05). Statistical analyses determined a two-way interaction between treatment and time for malate dehydrogenase, as shown in Figure 14C. Specific activities for lactate dehydrogenase and malate dehydrogenase are shown in Figure 14B,D.

## 4. Discussion

The pathogenic role of oxidative stress in neurodegenerative disease in relation to glutathione depletion has been well documented. Research demonstrates the contribution of oxidative stress to the energy crisis observed post-TBI. Studies show that the attenuation of oxidative stress experienced upon traumatic brain injury impedes the disruption of redox homeostasis, which can improve cellular and functional outcomes after injury. Given the complexity and heterogeneity of TBI, therapeutic approaches to combat its deleterious effects are still non-existent. However, the administration of chemical substances, such as GCEE, in experimental settings has been shown to increase intracellular glutathione levels, which in turn alleviates neurotoxic byproducts accumulated upon injury and offers therapeutic promise. We investigated the enzymatic activity of the energy-related proteins glyceraldehyde-3-phosphate dehydrogenase, pyruvate kinase, lactate dehydrogenase, aspartate aminotransferase, malate dehydrogenase, cytochrome C oxidase, and ATP synthase, which were found to be excessively nitrated in moderate TBI, and assessed the efficacy of GCEE as a potential therapeutic strategy in improving brain energy metabolism following moderate TBI [17].

Aspartate aminotransferase’s catalytic function is vital to amino acid metabolism. Impairments in AST activity may contribute to the increased concentration of glutamate observed following primary TBI [48,49]. Here we report increased enzymatic activity following the administration of GCEE 60 min post-TBI compared to saline-treated and sham rats. This may contribute to reducing amino acid dysregulation exhibited in traumatic brain injury via glutathione elevation [50]. The ability of GCEE to impede the cascade of ROS production resulting from glutamate-induced excitotoxicity and increased calcium influx may alleviate AST inactivation due to protein oxidation.

ATP synthase plays a key role in energy production and transduction in cells. Reduced energy metabolism as well as mitochondrial dysfunction have been subsequently observed in TBI, as well as other neurodegenerative diseases as a consequence of oxidative stress and damage. Reductions in ATP synthase activity are indicative of oxidative modifications incurred by proteins via increased ROS/RNS production after TBI, possibly resulting in impaired ATP production. The administration of GCEE following injury resulted in significant increases in ATP synthase activity, which suggests that GCEE is protective in reducing the oxidative modifications of ATP synthase experienced post traumatic brain injury.

Cytochrome C oxidase activity is an indicator of cellular oxidative capacity under normal physiological conditions [51]. Deficient CCO activity via protein dysfunction has been correlated with clinical and experimental manifestations of mitochondrial dysfunction resulting in TBI [52]. Reduced alterations in cytochrome C oxidase activity may directly affect cellular ATP levels and calcium homeostasis. The attenuation of oxidative stress via administration of GCEE may indirectly increase ATP levels in individuals suffering from traumatic brain injury by improving mitochondrial function, decreasing calcium influx, and decreasing susceptibility of cytochrome C to oxidative modifications.

Enolase and creatine kinase serve as important enzymes in the brain and are essential to energy metabolism. Enolase is an integral part of energy production through glycolysis. Creatine kinase provides rapid ATP buffering capacity, serving as an energy reservoir throughout the cell [53]. Enzymatic analysis results (Figure 11C and Figure 13A) demonstrated increased enzyme activity for both creatine kinase and enolase in samples treated with GCEE 30 min post-injury. Creatine kinase also showed increased enzyme activity in samples treated with GCEE 60 min post-injury. This indicates an increase in energy production in GCEE-treated samples via an increase in phosphoryl group transfer and phosphoenolpyruvate formation via creatine kinase and enolase activity, respectively. Saline-treated samples also showed increased enzyme activity, suggesting that enolase and creatine kinase might be upregulated during times of oxidative stress, as an increase in catalytic activity of an enzyme can be an indication of its concentration [54]. Although creatine kinase and enolase have been found inadequate as precise measures of injury severity, possible upregulation could provide important information on TBI, allowing for incidence of injury and GCEE efficacy [55,56,57]. Upregulation of enolase and creatine kinase may play a role in metabolic enzyme activity during TBI, so it is clear that function of these enzymes is essential during post-injury.

Glyceraldehyde-3-phosphate dehydrogenase is a multifunctional protein with glycolytic, sensory, and apoptotic activities. Studies demonstrate the functional role of glyceraldehyde-3-phosphate dehydrogenase in the progression of neurodegenerative disease, as impaired glycolytic function and increased pro-apoptotic function is observed in AD and HD, respectively [58,59,60]. ATP is produced via substrate-level phosphorylation. Impairment of GAPDH glycolytic activity in persons who have experienced TBI may contribute to increased concentrations of glyceraldehyde-3-phosphate and dihydroxyacetone, which give rise to methylgloxal, an oxidative metabolite associated with DNA and protein modification [61]. The mitochondrial binding of GAPDH leads to calcium uptake, matrix swelling, and the release of cytochrome C [62], which also occur in neurodegeneration. Attenuation of oxidative stress via administration of GCEE following traumatic brain injury may improve maintenance of glycolytic function while simultaneously reducing neuronal cell death commonly observed in TBI.

Lactate dehydrogenase is a biomarker of clinical interest in traumatic brain injury. The utilization of lactate as a substrate for gluconeogenesis ensures the perpetuation of glucose and energy metabolism in circumstances of limited glucose availability. Lowered glucose metabolism attributed to LDH dysfunction has been observed in AD, PD, and MCI [63,64]. Impairment in the catalytic activity of lactate dehydrogenase via oxidative modifications could result in reduced gluconeogenesis and the overproduction and/or accumulation of pyruvate [64]. It should be noted that lactate dehydrogenase activity was increased by saline administration. This was contrary to most enzymatic activities after TBI. However, LDH activity has been shown to be increased post incident due to an increase in lactate concentration, a hallmark of this disorder, which can be sustained up to 7 days after injury [65,66]. Inhibition of protein oxidative damage associated with TBI via administration of GCEE may increase LDH enzymatic activity, which may successively result in increased brain lactate levels, glucose production, NAD+ production, and energy metabolism.

Malate dehydrogenase has been shown to be oxidatively modified in persons with MCI [64], and its activity has been demonstrated to be elevated in persons with AD [37,67,68]. Impairments in the activity of MDH may result in reduced oxaloacetate and ATP production in persons with TBI. Increases in MDH activity may elevate oxaloacetate levels, which may in turn decrease the increasingly high levels of glutamate (and excitotoxicity) experienced after brain injury, as oxaloacetate has been demonstrated to scavenge glutamate [69]. Additionally, elevated malate dehydrogenase activity may also increase oxaloacetate levels and passage of reducing equivalents to ETC, which may profoundly increase the reduced ATP production exhibited in TBI-affected individuals. In this current study, we have demonstrated that the administration of GCEE significantly increases MDH activity in TBI rats, where treatment administered 60 min post-injury significantly increases activity compared to treatment administered at 30 min. The ability of GCEE to increase activity suggests its efficacy as a therapy to attenuate oxidative stress and increased MDH susceptibility to oxidative modification.

Evidence indicates reduced enzyme activity of PK in individuals with amnestic mild cognitive impairment (MCI), a predecessor condition frequently experienced upon development of Alzheimer’s disease, and posits that protein function is impaired by oxidative modification. Given its glycolytic nature, pyruvate kinase is vital to the production of energy in the brain, as pyruvate acts as an intermediate to continue cellular respiration. Oxidative modification of pyruvate kinase observed in individuals with MCI results in limited ATP production and altered ATP-dependent cellular activities, such as signal transduction and cell membrane potential maintenance [64]. Similar manifestations may be observed in persons with TBI, as pyruvate kinase has also been found to be oxidatively modified. The significantly increased enzymatic activity of PK upon the administration of GCEE 30 and 60 min compared to sham and saline-treated animals following traumatic brain injury suggests potential improvements in redox homeostasis and ATP production outcomes following TBI.

## 5. Conclusions

**Figure 15:**
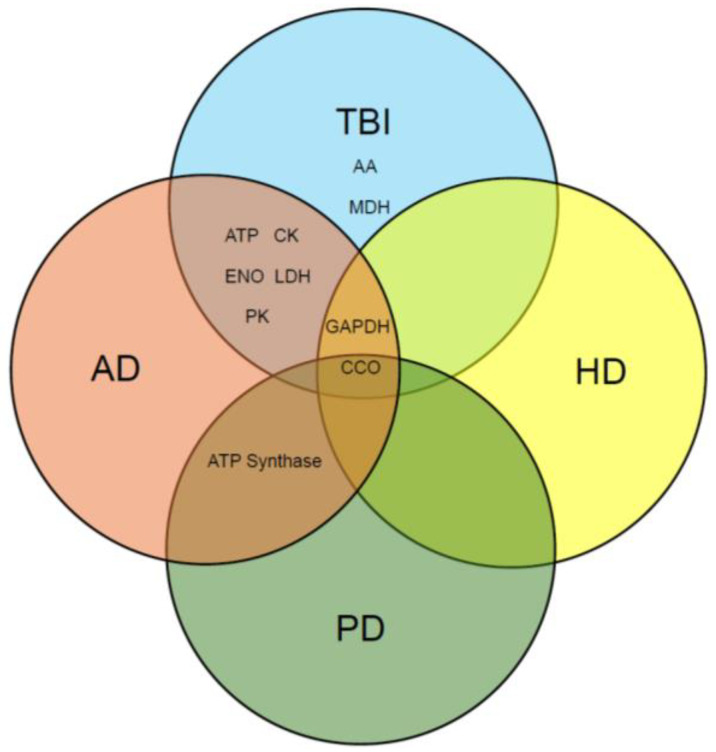
Venn diagram identifying proteins with reduced enzymatic activities observed in TBI, AD, HD, and PD.

In this study, we assessed the enzymatic activity of key energy-related proteins identified as being excessively nitrated. Oxidative modification of proteins contributes to the alterations in enzyme activities found in many neurodegenerative disorders. Slight reductions in the activities of some proteins were observed upon the administration of the vehicle following TBI, where pyruvate kinase and aspartate aminotransferase activity were reduced upon administration at 30 min, while ATP synthase activity was reduced at 30 and 60 min. Elevations in the activities of glyceraldehyde-3-phosphate dehydrogenase, pyruvate kinase, lactate dehydrogenase, aspartate aminotransferase, malate dehydrogenase, and cytochrome C oxidase upon administration of saline are suggestive of saline providing protection from protein oxidation incurred post-injury. The administration of saline solutions containing hydrogen, pyruvate, and lactate have been shown to diminish the effects of secondary brain injury by attenuating increased ROS/RNS production, enhancing bioenergetics, and improving neurological recovery, respectively [70]. All proteins in this current investigation were found to be upregulated, with significant elevations observed in the activities of ATP synthase, pyruvate kinase, and malate dehydrogenase. These exhibited the largest restorations by GCEE treatment and may be a new avenue of investigation for therapeutic targets. Given that the administration of GCEE attenuates the oxidative stress observed after TBI, elevations in the activities of proteins upon administration of the drug in the vehicle compared to only the vehicle suggest saline’s ability to enhance attenuation of oxidative stress. Trends in the activities indicated that administration of GCEE 60 min post-TBI led to the highest level of activity compared to treatment at 30 min, with significant observations of increased activity in pyruvate kinase, aspartate aminotransferase, and malate dehydrogenase. The treatment window was limited to one hour, which may show ideal efficacy if translated to a larger animal model. Time point expansion should be considered. Although statistically significant results were produced, a small sample size was used. A larger sample size may yield better findings. If this therapeutic is planned to be used for human trials, a different mode of delivery may be more efficient, as intraperitoneal injection may be challenging. This is the first study to investigate and compare enzymatic assays of these enzymes in TBI, AD, HD, and PD, therefore making it novel (Figure 15). As this treatment has been shown to restore enzymatic activity in moderate traumatic brain injury, it may be used for investigation in repeated head trauma. Taken together, these results suggest that GCEE is a promising potential therapeutic that increases the enzyme activity of several key energy-related proteins upon administration at one hour following traumatic brain injury.

## Figures and Tables

**Table 1:** Experimental design of enzymatic assay study.

Enzymatic Assays	Sham *n* = 6	TBI + Vehicle at 30 min *n* = 6	TBI + GCEE at 30 min *n* = 6	TBI + Vehicle at 60 min *n* = 6	TBI + GCEE at 60 min *n* = 6
AST	Yes	Yes	Yes	Yes	Yes
ATP synthase	Yes	Yes	Yes	Yes	Yes
CCO	Yes	Yes	Yes	Yes	Yes
CK	Yes	Yes	Yes	Yes	Yes
ENO	Yes	Yes	Yes	Yes	Yes
GAPDH	Yes	Yes	Yes	Yes	Yes
LDH	Yes	Yes	Yes	Yes	Yes
MDH	Yes	Yes	Yes	Yes	Yes
PK	Yes	Yes	Yes	Yes	Yes

## Data Availability

The data presented in this study are available on request from the corresponding author due to privacy reasons.
